# Supplementary material for: Inhibition of Biogenic Amines in Fermented Tilapia Surimi by Collaborative Fermentation of Latilactobacillus sakei and Pediococcus acidilactici
Source: Foods. 2024 Oct 17;13(20):3297. doi: 10.3390/foods13203297 (PMC11506939; doi:10.3390/foods13203297)
Supplement: Supplementary file 1 [file foods-13-03297-s001.zip › foods-3218829-supplementary.pdf]

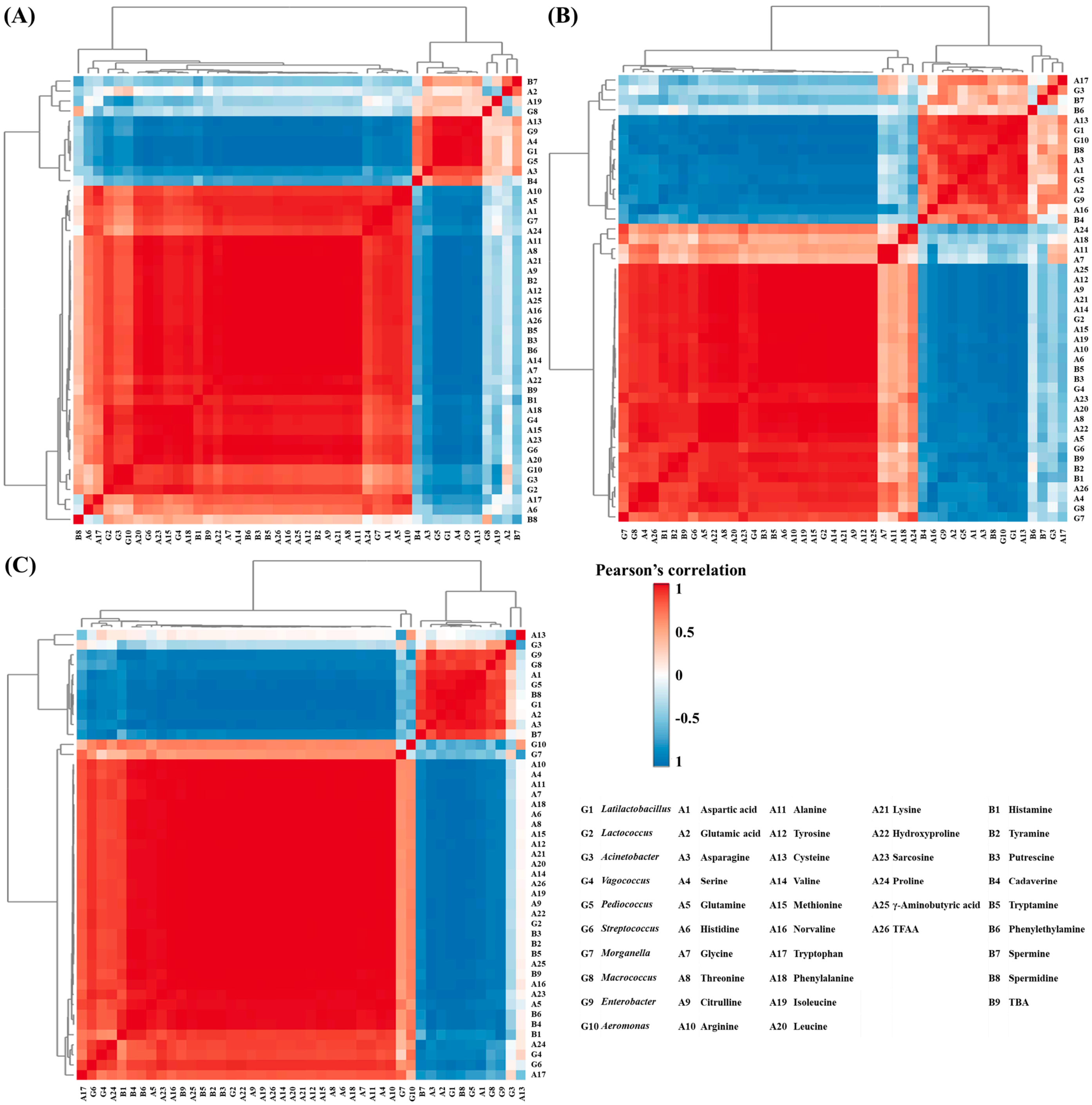

Figure S1 Correlation analysis among the core microbial genera, BAs, and FAAs in the group dimension in the (A) 18 h, (B) 30 h, and (C) 45 h periods.

Table S1 Changes in BAs (mg/kg) in the tilapia surimi during the fermentation process of the CM and CK groups

BAs	CK0	CK18	CK30	CK45	CM0	CM18	CM30	CM45
Histamine	0.82±0.06	38.66±1.17	44.18±1.16	39.09±1.43	0.81±0.04	28.67±2.73	29.34±3.14	35.67±0.81
Tyramine	ND	6.20±0.27	6.63±1.88	20.99±0.76	ND	0.11±0.02	0.11±0.03	0.19±0.01
Putrescine	0.29±0.09	13.06±0.26	14.28±0.42	27.23±0.96	0.30±0.03	ND	ND	ND
Cadaverine	1.37±0.56	14.60±0.23	21.16±0.56	34.82±1.39	1.53±0.25	16.58±1.61	23.11±1.31	25.08±0.58
Tryptamine	ND	1.59±0.01	2.26±0.09	3.95±0.09	ND	ND	ND	ND
Phenylethylamine	ND	8.86±0.13	1.93±0.23	5.64±0.18	ND	0.90±0.14	1.99±0.10	4.03±0.15
Spermine	3.60±0.76	1.40±0.02	1.57±0.06	1.37±0.14	3.51±0.03	2.09±0.56	3.20±1.40	1.67±0.05
Spermidine	1.30±0.10	0.79±0.06	0.39±0.04	0.13±0.12	1.37±0.03	0.72±0.15	1.52±0.19	1.45±0.08
TBA	7.38±0.83	85.15±1.67	92.41±4.30	133.24±5.03	7.52±0.27	49.08±3.94	59.27±3.30	68.11±1.65

“ND” indicates that the BA is not detected.

Table S2 Changes in FAAs (mg/kg) in the tilapia surimi during the fermentation process of the CM and CK groups

FAAs	CK0	CK18	CK30	CK45	CM0	CM18	CM30	CM45
Aspartic acid	0.023±0.001	0.017±0.002	0.022±0.001	0.054±0.001	0.025±0.001	0.010±0.000	0.025±0.001	0.058±0.001
Glutamic acid	0.106±0.006	0.066±0.001	0.060±0.002	0.065±0.005	0.113±0.004	0.066±0.002	0.083±0.005	0.095±0.001
Asparagine	0.003±0.000	0.003±0.000	0.003±0.000	0.004±0.000	0.002±0.000	0.003±0.000	0.005±0.000	0.006±0.001
Serine	0.036±0.001	0.014±0.000	0.034±0.003	0.058±0.001	0.045±0.001	0.031±0.001	0.029±0.002	0.039±0.002
Glutamine	0.113±0.004	0.093±0.002	0.093±0.001	0.095±0.002	0.127±0.002	0.081±0.003	0.079±0.003	0.079±0.002
Histidine	0.155±0.012	0.034±0.002	0.049±0.001	0.070±0.001	0.167±0.003	0.032±0.001	0.030±0.001	0.027±0.001
Glycine	1.965±0.044	2.087±0.003	2.110±0.012	2.021±0.022	1.937±0.030	1.952±0.009	2.049±0.135	1.714±0.009
Threonine	0.095±0.006	0.021±0.001	0.034±0.001	0.072±0.001	0.096±0.005	0.016±0.000	0.026±0.001	0.042±0.001
Citrulline	0.012±0.001	0.014±0.000	0.020±0.000	0.044±0.000	0.017±0.001	0.008±0.000	0.009±0.000	0.017±0.001
Arginine	0.015±0.001	0.003±0.000	0.055±0.001	0.021±0.000	0.006±0.001	0.002±0.000	0.007±0.001	0.010±0.001
Alanine	1.476±0.023	1.624±0.009	1.683±0.005	1.735±0.021	1.474±0.018	1.505±0.006	1.618±0.107	1.395±0.010
Tyrosine	0.022±0.002	0.041±0.001	0.060±0.005	0.062±0.002	0.020±0.001	0.014±0.000	0.027±0.001	0.041±0.001
Cysteine	0.794±0.003	0.003±0.000	0.004±0.001	0.012±0.003	0.769±0.007	0.007±0.000	0.010±0.001	0.012±0.003
Valine	0.052±0.003	0.047±0.001	0.104±0.001	0.231±0.002	0.057±0.001	0.028±0.000	0.048±0.003	0.068±0.001
Methionine	0.035±0.001	0.045±0.002	0.086±0.002	0.196±0.003	0.035±0.001	0.035±0.001	0.050±0.002	0.073±0.003
Norvaline	0.015±0.001	0.058±0.002	0.002±0.0010	0.021±0.001	0.016±0.004	0.044±0.002	0.006±0.002	0.012±0.001
Tryptophan	0.386±0.003	0.405±0.003	0.385±0.004	0.361±0.005	0.380±0.013	0.394±0.007	0.395±0.012	0.336±0.016
Phenylalanine	0.017±0.001	0.139±0.008	0.152±0.057	0.289±0.004	0.015±0.000	0.092±0.002	0.121±0.010	0.064±0.001
Isoleucine	0.035±0.002	0.041±0.005	0.080±0.001	0.173±0.002	0.062±0.004	0.044±0.001	0.055±0.002	0.067±0.005
Leucine	0.042±0.006	0.042±0.002	0.102±0.001	0.305±0.010	0.053±0.003	0.033±0.001	0.068±0.004	0.113±0.012
Lysine	0.133±0.011	0.168±0.007	0.238±0.002	0.422±0.004	0.143±0.002	0.067±0.001	0.097±0.007	0.129±0.001
Hydroxyproline	0.803±0.006	0.975±0.017	1.101±0.006	1.355±0.012	0.787±0.012	0.841±0.006	0.918±0.030	0.851±0.011
Sarcosine	0.105±0.002	0.132±0.003	0.139±0.006	0.183±0.010	0.109±0.002	0.103±0.002	0.107±0.001	0.104±0.003
Proline	0.049±0.002	0.037±0.005	0.036±0.006	0.042±0.009	0.047±0.001	0.027±0.002	0.030±0.002	0.025±0.002
γ-Aminobutyric acid	ND	0.130±0.002	0.185±0.011	0.347±0.004	ND	0.066±0.001	0.078±0.003	0.100±0.010
TFAA	6.488±0.128	6.239±0.028	6.834±0.046	8.241±0.064	6.505±0.068	5.501±0.027	5.969±0.323	5.475±0.046

“ND” indicates that the FAA is not detected.
